# 4-(4-Chloro­phen­yl)-2,6-bis­(1*H*-indol-3-yl)-1,4-dihydro­pyridine-3,5-dicarbo­nitrile ethanol monosolvate

**DOI:** 10.1107/S1600536812013906

**Published:** 2012-04-04

**Authors:** Song-Lei Zhu, Jun-Nian Zheng

**Affiliations:** a Key Laboratory of Biological Cancer Therapy, Xuzhou Medical College, Xuzhou 221004, People’s Republic of China

## Abstract

In the title compound, C_29_H_18_ClN_5_·C_2_H_6_O, the dihydro­pyridine ring adopts a strongly flattened envelope conformation, with a maximum deviation of 0.139 (2) Å from its best plane for the C*sp* ^3^ atom. The dihedral angles between the dihydro­pyridine ring plane and the two indole rings in positions 2 and 6 are 34.28 (5) and 40.50 (6)°, respectively. In turn, the benzene ring and the dihydro­pyridine ring are oriented at a dihedral angle of 74.69 (6)°. An intra­molecular C—H⋯Cl hydrogen bond occurs. In the crystal, mol­ecules are linked by N—H⋯N, N—H⋯O and O—H⋯N hydrogen bonds into layers parallel to (001). There are short C—H⋯Cl contacts between mol­ecules in neighboring layers.

## Related literature

For the biological activity of indole and 1,4-dihydro­pyridine derivatives, see: da Silva *et al.* (2001 ▶); Joshi & Chand (1982 ▶); Janis & Triggle (1983 ▶). For the synthesis of a series of bis­indoles derivatives of 1,4-dihydro­pyridine, see: Zhu *et al.* (2008 ▶).
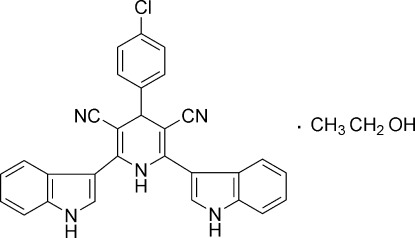


## Experimental

### 

#### Crystal data

C_29_H_18_ClN_5_·C_2_H_6_O
*M* *_r_* = 518.00
Triclinic, 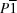

*a* = 9.2133 (17) Å
*b* = 11.611 (2) Å
*c* = 12.473 (2) Å
α = 87.714 (7)°
β = 83.297 (6)°
γ = 89.576 (7)°
*V* = 1324.1 (4) Å^3^
*Z* = 2
Mo *K*α radiation
μ = 0.18 mm^−1^
*T* = 193 K
0.55 × 0.36 × 0.15 mm

#### Data collection

Rigaku Mercury diffractometer
Absorption correction: multi-scan (*REQAB*; Jacobson, 1998 ▶) *T*_min_ = 0.787, *T*_max_ = 0.974
12971 measured reflections
4803 independent reflections
4095 reflections with *I* > 2σ(*I*)
*R*_int_ = 0.029

#### Refinement

*R*[*F* ^2^ > 2σ(*F* ^2^)] = 0.054
*wR*(*F* ^2^) = 0.119
*S* = 1.09
4803 reflections
346 parameters
H-atom parameters constrained
Δρ_max_ = 0.67 e Å^−3^
Δρ_min_ = −0.71 e Å^−3^

### 

Data collection: *CrystalClear* (Rigaku/MSC, 2001 ▶); cell refinement: *CrystalClear*; data reduction: *CrystalStructure* (Rigaku/MSC, 2004 ▶); program(s) used to solve structure: *SHELXS97* (Sheldrick, 2008 ▶); program(s) used to refine structure: *SHELXL97* (Sheldrick, 2008 ▶); molecular graphics: *ORTEPII* (Johnson, 1976 ▶) and *PLATON* (Spek, 2009 ▶); software used to prepare material for publication: *SHELXL97* and *PLATON*.

## Supplementary Material

Crystal structure: contains datablock(s) global, I. DOI: 10.1107/S1600536812013906/gk2473sup1.cif

Structure factors: contains datablock(s) I. DOI: 10.1107/S1600536812013906/gk2473Isup2.hkl

Supplementary material file. DOI: 10.1107/S1600536812013906/gk2473Isup3.cml

Additional supplementary materials:  crystallographic information; 3D view; checkCIF report

## Figures and Tables

**Table 1:** Hydrogen-bond geometry (Å, °)

*D*—H⋯*A*	*D*—H	H⋯*A*	*D*⋯*A*	*D*—H⋯*A*
C31—H31*A*⋯Cl1	0.99	2.83	3.571 (3)	132
C28—H28⋯Cl1^i^	0.95	2.79	3.520 (2)	135
N5—H5⋯O1^ii^	0.88	2.04	2.907 (2)	167
N2—H2⋯N4^iii^	0.88	2.18	2.989 (2)	153
N1—H1*A*⋯O1^iv^	0.88	2.04	2.834 (2)	150
O1—H1⋯N3^v^	0.84	1.96	2.791 (2)	172

Symmetry codes: (i) 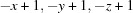; (ii) 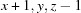; (iii) 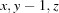; (iv) 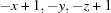; (v) 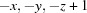.

**Fig. 1.**
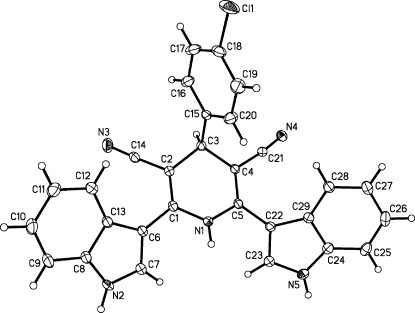
The molecular structure of the title molecule, with the atom-numbering scheme. Displacement ellipsoids are drawn at the 30% probability level. The solvent ethanol is not shown for clarity.

**Fig. 2.**
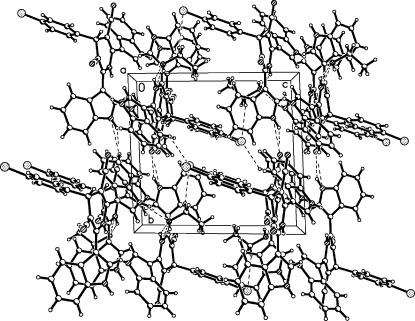
A packing diagram of the title compound. Hydrogen bonds are shown as dashed lines.

## supplementary crystallographic information

### Comment

Indole fragments are important moieties of a large number of natural products and medicinal agents (da Silva *et al.*, 2001). Compounds carrying the indole moiety exhibit antibacterial and fungicidal activities (Joshi & Chand, 1982). In addition, 1,4-dihydropyridine compounds are a well-known classe of calcium channel modulators for the treatment of cardiovascular diseases, for example Nifedipin, Felodipin are clinically useful as vasodilators and antihypertensive agents (Janis & Triggle, 1983). Due to the potent and diverse biological activities of indole and 1,4-dihydropyridine derivatives, we investigated a simple and efficient protocol for synthesis of a series of bisindoles derivatives containing 1,4-dihydropyridine units (Zhu *et al.*, 2008). Herein, we report the crystal structure of the title compound.

In the title molecule (Fig. 1), atoms of the newly formed 1,4-dihydropyridine ring A (N1, C1-C5) are nearly planar, with the maximum deviation of 0.139 (2) Å. The dihedral angles between ring A with attached two indole rings B (N2, C6-C13) and C (N5, C22-C29) are 34.28 (5) and 40.50 (6)°, respectively. Ring A and the benzene ring D (C15-C20) are oriented at a dihedral angle of 74.69 (6)°.

In the crystal, intermolecular N-H···N, N-H···O and O-H···N hydrogen bonds link the molecules into layers parallel to (0 0 1) (Table 1, Fig. 2). There are short C-H···Cl contacts between the molecules from neighboring layers.

### Experimental

The title compound was prepared by the reaction of 4-chlorobenzaldehyde (1 mmol), 3-cyanoacetyl indole (2 mmol), ammonium acetate (5 mmol) in glycol solvent (3 mL) under microwave irradiation condition. After irradiating for 8 mins at 413 K, the reaction mixture was cooled and washed with small amount of ethanol. The crude product was filtered and single crystals of the title compound were obtained from ethanol solution by slow evaporation at room temperature (yield: 75%, m.p. > 573 K).

### Refinement

H atoms were positioned geometrically, with N-H = 0.88 Å , O-H = 0.84 Å (for OH), and C-H =0.95, 0.98, 0.99, 1.00 Å for aromatic, methyl, methylene, and methyne H, respectively, and constrained to ride on their parent atoms with U_iso_(H) = x U_eq_(C,N,O), where x = 1.5 for methyl and hydroxyl H, x = 1.2 for all other H atoms.

### Crystal data

**Table:**

C_29_H_18_ClN_5_·C_2_H_6_O	*Z* = 2
*M**_r_* = 518.00	*F*(000) = 540
Triclinic, *P*1	*D*_x_ = 1.299 Mg m^−^^3^
Hall symbol: -P 1	Melting point > 573 K
*a* = 9.2133 (17) Å	Mo *K*α radiation, λ = 0.71070 Å
*b* = 11.611 (2) Å	Cell parameters from 4662 reflections
*c* = 12.473 (2) Å	θ = 3.1–25.3°
α = 87.714 (7)°	µ = 0.18 mm^−^^1^
β = 83.297 (6)°	*T* = 193 K
γ = 89.576 (7)°	Block, colorless
*V* = 1324.1 (4) Å^3^	0.55 × 0.36 × 0.15 mm

### Data collection

**Table:**

Rigaku Mercury diffractometer	4803 independent reflections
Radiation source: fine-focus sealed tube	4095 reflections with *I* > 2σ(*I*)
Graphite monochromator	*R*_int_ = 0.029
Detector resolution: 7.31 pixels mm^-1^	θ_max_ = 25.4°, θ_min_ = 3.1°
ω scans	*h* = −11→10
Absorption correction: multi-scan (*REQAB*; Jacobson, 1998)	*k* = −13→13
*T*_min_ = 0.787, *T*_max_ = 0.974	*l* = −14→15
12971 measured reflections	

### Refinement

**Table:**

Refinement on *F*^2^	Primary atom site location: structure-invariant direct methods
Least-squares matrix: full	Secondary atom site location: difference Fourier map
*R*[*F*^2^ > 2σ(*F*^2^)] = 0.054	Hydrogen site location: inferred from neighbouring sites
*wR*(*F*^2^) = 0.119	H-atom parameters constrained
*S* = 1.09	*w* = 1/[σ^2^(*F*_o_^2^) + (0.0408*P*)^2^ + 0.8429*P*] where *P* = (*F*_o_^2^ + 2*F*_c_^2^)/3
4803 reflections	(Δ/σ)_max_ < 0.001
346 parameters	Δρ_max_ = 0.67 e Å^−^^3^
0 restraints	Δρ_min_ = −0.71 e Å^−^^3^

### Special details

**Table:**

Geometry. All esds (except the esd in the dihedral angle between two l.s. planes) are estimated using the full covariance matrix. The cell esds are taken into account individually in the estimation of esds in distances, angles and torsion angles; correlations between esds in cell parameters are only used when they are defined by crystal symmetry. An approximate (isotropic) treatment of cell esds is used for estimating esds involving l.s. planes.
Refinement. Refinement of F^2^ against ALL reflections. The weighted R-factor wR and goodness of fit S are based on F^2^, conventional R-factors R are based on F, with F set to zero for negative F^2^. The threshold expression of F^2^ > 2sigma(F^2^) is used only for calculating R-factors(gt) etc. and is not relevant to the choice of reflections for refinement. R-factors based on F^2^ are statistically about twice as large as those based on F, and R- factors based on ALL data will be even larger.

### Fractional atomic coordinates and isotropic or equivalent isotropic displacement parameters (Å^2^)

**Table:**

	*x*	*y*	*z*	*U*_iso_*/*U*_eq_	
Cl1	0.31431 (10)	0.42027 (6)	0.66333 (5)	0.0645 (3)	
O1	0.16731 (15)	0.06664 (12)	0.79527 (12)	0.0305 (4)	
H1	0.0989	0.0202	0.7909	0.046*	
N1	0.59953 (18)	0.07570 (14)	0.14897 (14)	0.0249 (4)	
H1A	0.6694	0.0235	0.1421	0.030*	
N2	0.46514 (19)	−0.27027 (14)	0.14306 (15)	0.0308 (4)	
H2	0.4969	−0.3323	0.1092	0.037*	
N3	0.0789 (2)	0.06862 (17)	0.21675 (19)	0.0442 (5)	
N4	0.5715 (2)	0.48918 (15)	0.10560 (15)	0.0324 (4)	
N5	0.98636 (19)	0.19122 (16)	−0.04096 (15)	0.0325 (4)	
H5	1.0473	0.1647	−0.0940	0.039*	
C1	0.4581 (2)	0.03934 (17)	0.17981 (16)	0.0233 (4)	
C2	0.3512 (2)	0.12010 (17)	0.20019 (16)	0.0240 (4)	
C3	0.3834 (2)	0.24738 (16)	0.21036 (16)	0.0231 (4)	
H3	0.3113	0.2925	0.1715	0.028*	
C4	0.5345 (2)	0.27287 (16)	0.15183 (16)	0.0219 (4)	
C5	0.6369 (2)	0.19035 (16)	0.12830 (16)	0.0223 (4)	
C6	0.4366 (2)	−0.08497 (17)	0.18466 (16)	0.0245 (4)	
C7	0.5099 (2)	−0.16103 (17)	0.11543 (18)	0.0281 (5)	
H7	0.5813	−0.1400	0.0569	0.034*	
C8	0.3627 (2)	−0.26903 (18)	0.23208 (18)	0.0294 (5)	
C9	0.2898 (3)	−0.36089 (19)	0.2901 (2)	0.0384 (6)	
H9	0.3053	−0.4381	0.2688	0.046*	
C10	0.1950 (3)	−0.3353 (2)	0.3789 (2)	0.0458 (6)	
H10	0.1423	−0.3959	0.4191	0.055*	
C11	0.1741 (3)	−0.2218 (2)	0.4119 (2)	0.0439 (6)	
H11	0.1090	−0.2072	0.4748	0.053*	
C12	0.2463 (2)	−0.13097 (19)	0.35472 (19)	0.0346 (5)	
H12	0.2318	−0.0544	0.3779	0.041*	
C13	0.3412 (2)	−0.15357 (17)	0.26201 (17)	0.0269 (5)	
C14	0.2016 (2)	0.08876 (17)	0.21017 (18)	0.0295 (5)	
C15	0.3656 (2)	0.28430 (16)	0.32702 (16)	0.0243 (4)	
C16	0.2305 (2)	0.32231 (19)	0.37341 (19)	0.0342 (5)	
H16	0.1487	0.3211	0.3336	0.041*	
C17	0.2140 (3)	0.3620 (2)	0.4777 (2)	0.0427 (6)	
H17	0.1211	0.3875	0.5094	0.051*	
C18	0.3327 (3)	0.3643 (2)	0.53466 (19)	0.0400 (6)	
C19	0.4659 (3)	0.3244 (2)	0.4922 (2)	0.0463 (6)	
H19	0.5465	0.3240	0.5332	0.056*	
C20	0.4817 (2)	0.2842 (2)	0.38802 (19)	0.0372 (6)	
H20	0.5741	0.2562	0.3581	0.045*	
C21	0.5614 (2)	0.39169 (18)	0.12352 (16)	0.0247 (4)	
C22	0.7857 (2)	0.20992 (17)	0.07727 (16)	0.0239 (4)	
C23	0.8523 (2)	0.14721 (18)	−0.00612 (18)	0.0302 (5)	
H23	0.8105	0.0825	−0.0351	0.036*	
C24	1.0120 (2)	0.28385 (17)	0.01974 (17)	0.0276 (5)	
C25	1.1329 (2)	0.35680 (19)	0.01216 (19)	0.0339 (5)	
H25	1.2139	0.3477	−0.0414	0.041*	
C26	1.1297 (2)	0.4423 (2)	0.0853 (2)	0.0371 (6)	
H26	1.2094	0.4943	0.0814	0.045*	
C27	1.0116 (2)	0.45460 (19)	0.16554 (19)	0.0343 (5)	
H27	1.0144	0.5130	0.2164	0.041*	
C28	0.8913 (2)	0.38358 (18)	0.17213 (18)	0.0293 (5)	
H28	0.8118	0.3927	0.2269	0.035*	
C29	0.8885 (2)	0.29768 (17)	0.09657 (16)	0.0244 (4)	
C30	0.2484 (5)	0.0453 (3)	0.6042 (3)	0.0876 (12)	
H30A	0.3331	−0.0004	0.6213	0.131*	
H30B	0.2738	0.0898	0.5363	0.131*	
H30C	0.1667	−0.0063	0.5967	0.131*	
C31	0.2053 (3)	0.1251 (2)	0.6923 (2)	0.0435 (6)	
H31A	0.2872	0.1784	0.6978	0.052*	
H31B	0.1207	0.1718	0.6739	0.052*	

### Atomic displacement parameters (Å^2^)

**Table:**

	*U*^11^	*U*^22^	*U*^33^	*U*^12^	*U*^13^	*U*^23^
Cl1	0.1068 (6)	0.0464 (4)	0.0364 (4)	−0.0072 (4)	0.0148 (4)	−0.0185 (3)
O1	0.0256 (8)	0.0288 (8)	0.0366 (9)	−0.0044 (6)	−0.0005 (7)	−0.0018 (7)
N1	0.0224 (9)	0.0175 (9)	0.0338 (10)	−0.0003 (7)	0.0010 (7)	−0.0021 (7)
N2	0.0369 (10)	0.0176 (9)	0.0376 (11)	−0.0010 (8)	0.0001 (9)	−0.0083 (8)
N3	0.0285 (11)	0.0335 (11)	0.0708 (16)	−0.0054 (9)	−0.0083 (10)	0.0016 (10)
N4	0.0356 (10)	0.0219 (10)	0.0391 (11)	−0.0013 (8)	−0.0014 (8)	−0.0011 (8)
N5	0.0287 (10)	0.0360 (11)	0.0306 (10)	0.0005 (8)	0.0078 (8)	−0.0060 (8)
C1	0.0253 (10)	0.0214 (10)	0.0237 (10)	−0.0029 (8)	−0.0040 (8)	−0.0024 (8)
C2	0.0240 (10)	0.0215 (10)	0.0267 (11)	−0.0020 (8)	−0.0025 (8)	−0.0025 (8)
C3	0.0218 (10)	0.0193 (10)	0.0282 (11)	−0.0003 (8)	−0.0036 (8)	−0.0001 (8)
C4	0.0247 (10)	0.0189 (10)	0.0220 (10)	−0.0023 (8)	−0.0014 (8)	−0.0029 (8)
C5	0.0244 (10)	0.0199 (10)	0.0227 (10)	−0.0029 (8)	−0.0027 (8)	−0.0027 (8)
C6	0.0268 (10)	0.0193 (10)	0.0277 (11)	−0.0022 (8)	−0.0045 (9)	−0.0021 (8)
C7	0.0293 (11)	0.0233 (11)	0.0316 (12)	−0.0028 (9)	−0.0024 (9)	−0.0026 (9)
C8	0.0276 (11)	0.0226 (11)	0.0381 (13)	−0.0037 (9)	−0.0041 (10)	−0.0030 (9)
C9	0.0397 (13)	0.0215 (11)	0.0533 (16)	−0.0061 (10)	−0.0037 (12)	0.0010 (10)
C10	0.0396 (14)	0.0317 (13)	0.0630 (18)	−0.0080 (11)	0.0043 (13)	0.0099 (12)
C11	0.0400 (14)	0.0406 (14)	0.0467 (15)	−0.0004 (11)	0.0108 (12)	0.0053 (12)
C12	0.0372 (12)	0.0260 (12)	0.0390 (13)	−0.0005 (10)	0.0027 (10)	−0.0035 (10)
C13	0.0272 (11)	0.0217 (11)	0.0321 (12)	−0.0019 (8)	−0.0046 (9)	−0.0016 (9)
C14	0.0303 (12)	0.0194 (11)	0.0391 (13)	−0.0004 (9)	−0.0044 (10)	−0.0032 (9)
C15	0.0277 (11)	0.0155 (10)	0.0285 (11)	−0.0008 (8)	0.0024 (9)	−0.0019 (8)
C16	0.0304 (12)	0.0340 (13)	0.0360 (13)	0.0044 (10)	0.0045 (10)	0.0002 (10)
C17	0.0460 (15)	0.0339 (13)	0.0423 (15)	0.0102 (11)	0.0186 (12)	0.0003 (11)
C18	0.0579 (16)	0.0286 (12)	0.0306 (13)	−0.0058 (11)	0.0100 (12)	−0.0087 (10)
C19	0.0477 (15)	0.0599 (17)	0.0325 (14)	−0.0078 (13)	−0.0061 (12)	−0.0122 (12)
C20	0.0297 (12)	0.0491 (15)	0.0331 (13)	0.0028 (10)	−0.0023 (10)	−0.0109 (11)
C21	0.0250 (11)	0.0252 (12)	0.0237 (11)	−0.0005 (9)	−0.0009 (9)	−0.0037 (8)
C22	0.0256 (10)	0.0198 (10)	0.0253 (11)	0.0000 (8)	0.0011 (9)	−0.0007 (8)
C23	0.0290 (11)	0.0269 (11)	0.0342 (12)	−0.0029 (9)	0.0007 (10)	−0.0055 (9)
C24	0.0270 (11)	0.0253 (11)	0.0295 (12)	0.0003 (9)	−0.0018 (9)	0.0061 (9)
C25	0.0246 (11)	0.0359 (13)	0.0396 (13)	−0.0017 (10)	−0.0013 (10)	0.0118 (10)
C26	0.0295 (12)	0.0320 (13)	0.0513 (15)	−0.0088 (10)	−0.0139 (11)	0.0101 (11)
C27	0.0373 (13)	0.0265 (12)	0.0417 (14)	−0.0033 (10)	−0.0153 (11)	−0.0006 (10)
C28	0.0298 (11)	0.0260 (11)	0.0329 (12)	−0.0004 (9)	−0.0063 (10)	−0.0027 (9)
C29	0.0259 (11)	0.0210 (10)	0.0261 (11)	0.0006 (8)	−0.0037 (9)	0.0033 (8)
C30	0.141 (4)	0.076 (2)	0.0404 (18)	−0.016 (2)	0.015 (2)	−0.0096 (16)
C31	0.0500 (15)	0.0415 (14)	0.0389 (14)	−0.0043 (12)	−0.0071 (12)	0.0063 (11)

### Geometric parameters (Å, º)

**Table:**

Cl1—C18	1.745 (2)	C11—C12	1.380 (3)
O1—C31	1.435 (3)	C11—H11	0.9500
O1—H1	0.8400	C12—C13	1.398 (3)
N1—C1	1.379 (3)	C12—H12	0.9500
N1—C5	1.384 (2)	C15—C20	1.383 (3)
N1—H1A	0.8800	C15—C16	1.387 (3)
N2—C7	1.355 (3)	C16—C17	1.388 (3)
N2—C8	1.371 (3)	C16—H16	0.9500
N2—H2	0.8800	C17—C18	1.374 (4)
N3—C14	1.148 (3)	C17—H17	0.9500
N4—C21	1.147 (3)	C18—C19	1.364 (4)
N5—C23	1.357 (3)	C19—C20	1.389 (3)
N5—C24	1.377 (3)	C19—H19	0.9500
N5—H5	0.8800	C20—H20	0.9500
C1—C2	1.364 (3)	C22—C23	1.376 (3)
C1—C6	1.456 (3)	C22—C29	1.443 (3)
C2—C14	1.418 (3)	C23—H23	0.9500
C2—C3	1.523 (3)	C24—C25	1.396 (3)
C3—C4	1.520 (3)	C24—C29	1.412 (3)
C3—C15	1.523 (3)	C25—C26	1.373 (3)
C3—H3	1.0000	C25—H25	0.9500
C4—C5	1.356 (3)	C26—C27	1.400 (3)
C4—C21	1.427 (3)	C26—H26	0.9500
C5—C22	1.457 (3)	C27—C28	1.379 (3)
C6—C7	1.377 (3)	C27—H27	0.9500
C6—C13	1.445 (3)	C28—C29	1.402 (3)
C7—H7	0.9500	C28—H28	0.9500
C8—C9	1.396 (3)	C30—C31	1.483 (4)
C8—C13	1.412 (3)	C30—H30A	0.9800
C9—C10	1.369 (4)	C30—H30B	0.9800
C9—H9	0.9500	C30—H30C	0.9800
C10—C11	1.401 (4)	C31—H31A	0.9900
C10—H10	0.9500	C31—H31B	0.9900
			
C31—O1—H1	109.5	C20—C15—C3	121.96 (18)
C1—N1—C5	123.19 (16)	C16—C15—C3	119.59 (19)
C1—N1—H1A	118.4	C15—C16—C17	120.4 (2)
C5—N1—H1A	118.4	C15—C16—H16	119.8
C7—N2—C8	109.32 (17)	C17—C16—H16	119.8
C7—N2—H2	125.3	C18—C17—C16	119.6 (2)
C8—N2—H2	125.3	C18—C17—H17	120.2
C23—N5—C24	109.29 (17)	C16—C17—H17	120.2
C23—N5—H5	125.4	C19—C18—C17	121.2 (2)
C24—N5—H5	125.4	C19—C18—Cl1	119.1 (2)
C2—C1—N1	118.81 (18)	C17—C18—Cl1	119.73 (19)
C2—C1—C6	125.68 (18)	C18—C19—C20	119.0 (2)
N1—C1—C6	115.49 (17)	C18—C19—H19	120.5
C1—C2—C14	120.68 (18)	C20—C19—H19	120.5
C1—C2—C3	122.97 (17)	C15—C20—C19	121.4 (2)
C14—C2—C3	116.33 (17)	C15—C20—H20	119.3
C4—C3—C2	108.33 (16)	C19—C20—H20	119.3
C4—C3—C15	113.00 (16)	N4—C21—C4	174.1 (2)
C2—C3—C15	112.95 (16)	C23—C22—C29	106.34 (17)
C4—C3—H3	107.4	C23—C22—C5	124.24 (18)
C2—C3—H3	107.4	C29—C22—C5	129.35 (18)
C15—C3—H3	107.4	N5—C23—C22	110.18 (18)
C5—C4—C21	122.03 (18)	N5—C23—H23	124.9
C5—C4—C3	123.36 (17)	C22—C23—H23	124.9
C21—C4—C3	114.61 (17)	N5—C24—C25	129.8 (2)
C4—C5—N1	119.10 (17)	N5—C24—C29	107.72 (18)
C4—C5—C22	125.91 (18)	C25—C24—C29	122.5 (2)
N1—C5—C22	114.93 (17)	C26—C25—C24	117.2 (2)
C7—C6—C13	106.43 (17)	C26—C25—H25	121.4
C7—C6—C1	125.15 (19)	C24—C25—H25	121.4
C13—C6—C1	128.39 (18)	C25—C26—C27	121.5 (2)
N2—C7—C6	110.08 (19)	C25—C26—H26	119.3
N2—C7—H7	125.0	C27—C26—H26	119.3
C6—C7—H7	125.0	C28—C27—C26	121.3 (2)
N2—C8—C9	129.4 (2)	C28—C27—H27	119.3
N2—C8—C13	108.17 (18)	C26—C27—H27	119.3
C9—C8—C13	122.4 (2)	C27—C28—C29	118.8 (2)
C10—C9—C8	117.3 (2)	C27—C28—H28	120.6
C10—C9—H9	121.3	C29—C28—H28	120.6
C8—C9—H9	121.3	C28—C29—C24	118.59 (19)
C9—C10—C11	121.5 (2)	C28—C29—C22	134.93 (19)
C9—C10—H10	119.2	C24—C29—C22	106.45 (17)
C11—C10—H10	119.2	C31—C30—H30A	109.5
C12—C11—C10	121.1 (2)	C31—C30—H30B	109.5
C12—C11—H11	119.4	H30A—C30—H30B	109.5
C10—C11—H11	119.4	C31—C30—H30C	109.5
C11—C12—C13	118.9 (2)	H30A—C30—H30C	109.5
C11—C12—H12	120.6	H30B—C30—H30C	109.5
C13—C12—H12	120.6	O1—C31—C30	113.1 (2)
C12—C13—C8	118.70 (19)	O1—C31—H31A	109.0
C12—C13—C6	135.23 (19)	C30—C31—H31A	109.0
C8—C13—C6	105.99 (18)	O1—C31—H31B	109.0
N3—C14—C2	176.8 (2)	C30—C31—H31B	109.0
C20—C15—C16	118.4 (2)	H31A—C31—H31B	107.8
			
C5—N1—C1—C2	−5.9 (3)	C1—C6—C13—C12	−2.5 (4)
C5—N1—C1—C6	172.47 (17)	C7—C6—C13—C8	−0.9 (2)
N1—C1—C2—C14	166.92 (19)	C1—C6—C13—C8	−179.07 (19)
C6—C1—C2—C14	−11.3 (3)	C4—C3—C15—C20	−30.7 (3)
N1—C1—C2—C3	−11.3 (3)	C2—C3—C15—C20	92.7 (2)
C6—C1—C2—C3	170.48 (18)	C4—C3—C15—C16	147.46 (19)
C1—C2—C3—C4	22.5 (3)	C2—C3—C15—C16	−89.1 (2)
C14—C2—C3—C4	−155.86 (18)	C20—C15—C16—C17	1.7 (3)
C1—C2—C3—C15	−103.5 (2)	C3—C15—C16—C17	−176.58 (19)
C14—C2—C3—C15	78.2 (2)	C15—C16—C17—C18	0.4 (3)
C2—C3—C4—C5	−19.9 (3)	C16—C17—C18—C19	−2.4 (4)
C15—C3—C4—C5	106.0 (2)	C16—C17—C18—Cl1	177.11 (18)
C2—C3—C4—C21	160.43 (17)	C17—C18—C19—C20	2.1 (4)
C15—C3—C4—C21	−73.6 (2)	Cl1—C18—C19—C20	−177.4 (2)
C21—C4—C5—N1	−174.08 (18)	C16—C15—C20—C19	−1.9 (3)
C3—C4—C5—N1	6.3 (3)	C3—C15—C20—C19	176.2 (2)
C21—C4—C5—C22	2.9 (3)	C18—C19—C20—C15	0.1 (4)
C3—C4—C5—C22	−176.79 (18)	C4—C5—C22—C23	−134.1 (2)
C1—N1—C5—C4	8.5 (3)	N1—C5—C22—C23	43.0 (3)
C1—N1—C5—C22	−168.82 (18)	C4—C5—C22—C29	42.3 (3)
C2—C1—C6—C7	142.7 (2)	N1—C5—C22—C29	−140.6 (2)
N1—C1—C6—C7	−35.6 (3)	C24—N5—C23—C22	0.6 (3)
C2—C1—C6—C13	−39.5 (3)	C29—C22—C23—N5	−1.4 (2)
N1—C1—C6—C13	142.3 (2)	C5—C22—C23—N5	175.73 (19)
C8—N2—C7—C6	−0.8 (2)	C23—N5—C24—C25	−178.7 (2)
C13—C6—C7—N2	1.1 (2)	C23—N5—C24—C29	0.4 (2)
C1—C6—C7—N2	179.30 (18)	N5—C24—C25—C26	−179.1 (2)
C7—N2—C8—C9	−178.1 (2)	C29—C24—C25—C26	1.9 (3)
C7—N2—C8—C13	0.2 (2)	C24—C25—C26—C27	1.2 (3)
N2—C8—C9—C10	178.0 (2)	C25—C26—C27—C28	−2.1 (3)
C13—C8—C9—C10	−0.2 (3)	C26—C27—C28—C29	0.0 (3)
C8—C9—C10—C11	−1.3 (4)	C27—C28—C29—C24	2.9 (3)
C9—C10—C11—C12	1.3 (4)	C27—C28—C29—C22	−179.7 (2)
C10—C11—C12—C13	0.3 (4)	N5—C24—C29—C28	176.82 (18)
C11—C12—C13—C8	−1.8 (3)	C25—C24—C29—C28	−3.9 (3)
C11—C12—C13—C6	−177.9 (2)	N5—C24—C29—C22	−1.2 (2)
N2—C8—C13—C12	−176.76 (19)	C25—C24—C29—C22	177.99 (19)
C9—C8—C13—C12	1.7 (3)	C23—C22—C29—C28	−176.0 (2)
N2—C8—C13—C6	0.5 (2)	C5—C22—C29—C28	7.1 (4)
C9—C8—C13—C6	178.9 (2)	C23—C22—C29—C24	1.6 (2)
C7—C6—C13—C12	175.6 (2)	C5—C22—C29—C24	−175.3 (2)

### Hydrogen-bond geometry (Å, º)

**Table:**

*D*—H···*A*	*D*—H	H···*A*	*D*···*A*	*D*—H···*A*
C31—H31*A*···Cl1	0.99	2.83	3.571 (3)	132
C28—H28···Cl1^i^	0.95	2.79	3.520 (2)	135
N5—H5···O1^ii^	0.88	2.04	2.907 (2)	167
N2—H2···N4^iii^	0.88	2.18	2.989 (2)	153
N1—H1*A*···O1^iv^	0.88	2.04	2.834 (2)	150
O1—H1···N3^v^	0.84	1.96	2.791 (2)	172

Symmetry codes: (i) −*x*+1, −*y*+1, −*z*+1; (ii) *x*+1, *y*, *z*−1; (iii) *x*, *y*−1, *z*; (iv) −*x*+1, −*y*, −*z*+1; (v) −*x*, −*y*, −*z*+1.
